# Nanopore sequencing as a revolutionary diagnostic tool for porcine viral enteric disease complexes identifies porcine kobuvirus as an important enteric virus

**DOI:** 10.1038/s41598-018-28180-9

**Published:** 2018-06-29

**Authors:** Sebastiaan Theuns, Bert Vanmechelen, Quinten Bernaert, Ward Deboutte, Marilou Vandenhole, Leen Beller, Jelle Matthijnssens, Piet Maes, Hans J. Nauwynck

**Affiliations:** 1 Ghent University, Faculty of Veterinary Medicine, Department of Virology, Parasitology and Immunology, Laboratory of Virology, Merelbeke, Belgium; 2 KU Leuven - University of Leuven, Department of Microbiology and Immunology, Laboratory of Clinical Virology, Rega Institute for Medical Research, Leuven, Belgium; 3 KU Leuven - University of Leuven, Department of Microbiology and Immunology, Laboratory of Viral Metagenomics, Rega Institute for Medical Research, Leuven, Belgium

**Keywords:** Infectious-disease diagnostics, Rotavirus, Bacteriophages, Viral epidemiology, Metagenomics

## Abstract

Enteric diseases in swine are often caused by different pathogens and thus metagenomics are a useful tool for diagnostics. The capacities of nanopore sequencing for viral diagnostics were investigated here. First, cell culture-grown porcine epidemic diarrhea virus and rotavirus A were pooled and sequenced on a MinION. Reads were already detected at 7 seconds after start of sequencing, resulting in high sequencing depths (19.2 to 103.5X) after 3 h. Next, diarrheic feces of a one-week-old piglet was analyzed. Almost all reads (99%) belonged to bacteriophages, which may have reshaped the piglet’s microbiome. Contigs matched *Bacteroides*, *Escherichia* and *Enterococcus* phages. Moreover, porcine kobuvirus was discovered in the feces for the first time in Belgium. Suckling piglets shed kobuvirus from one week of age, but an association between peak of viral shedding (10^6.42^–10^7.01^ copies/swab) and diarrheic signs was not observed during a follow-up study. Retrospective analysis showed the widespread (n = 25, 56.8% positive) of genetically moderately related kobuviruses among Belgian diarrheic piglets. MinION enables rapid detection of enteric viruses. Such new methodologies will change diagnostics, but more extensive validations should be conducted. The true enteric pathogenicity of porcine kobuvirus should be questioned, while its subclinical importance cannot be excluded.

## Introduction

Swine health is continuously challenged by viral infections causing enteric, respiratory, reproductive and general systemic diseases. The etiological roles of rotaviruses and coronaviruses (transmissible gastroenteritis virus (TGEV) and porcine epidemic diarrhea virus (PEDV)) in the pathogenesis of piglet diarrhea have been widely accepted^1,2^. However, several novel and poorly characterized viruses are often found in feces of pigs. The clinical importance has not been addressed for most of them and they have not been included in the lists of differential diagnosis for swine veterinarians. Logically, diagnostics are not available for such viruses. Shotgun viral metagenomics allows simultaneous identification of known and novel viruses present in a porcine diagnostic sample. Many studies have already been conducted to elucidate the composition of the fecal virome of pigs, identifying several novel or poorly characterized viruses such as kobuviruses, orthoreoviruses, astroviruses, enteroviruses, circular DNA viruses, sapoviruses, bocaviruses, picobirnaviruses, posaviruses, sapeloviruses, teschoviruses and others^3–10^. Studies conducted in pigs from Belgium and the United States also revealed the global spread of a viral inter-family recombinant enterovirus carrying a torovirus-like gene insertion^10–12^. It is clear that viral metagenomics is a valuable asset for diagnostics in pigs, leading to discovery of novel viruses and identification of porcine viral enteric disease complexes. Although standardized procedures have been developed to study viral metagenomes in fecal samples, they still require an extensive sample preparation, including random or targeted pre-amplification of viral genomes present in the sample^13^. Most sequencing platforms still require capital investments and high sample turnover rates to be cost-effective. Performing the necessary analyses often results in long time periods between sample arrival and diagnostic reporting, since results can only be processed after finishing the sequencing run. Third-generation sequencing using MinION (Oxford Nanopore Technologies, ONT) might be a useful and affordable diagnostic tool for swine veterinary medicine as it allows rapid sample preparation and real-time sequence analysis. The flowcells used for sequencing consist of a membrane containing multiple CsgG nanopore proteins from *Escherichia coli*^14^. An ion current is established through this pore resulting in typical current changes upon passage of specific nucleotides. This signal is converted into a nucleotide sequence by computational algorithms (basecalling). Since the release of MinION technology, major advances have been made in terms of the number and the quality of reads generated^15^. In the field of virology, the technology has mainly been applied in human medicine. Using nanopore sequencing, it was possible to distinguish three poxviruses with 98% nucleotide similarity at strain level^16^. MinION has also been used as a diagnostic tool during recent Ebolavirus outbreaks in West Africa, allowing fast on-site characterization of circulating strains^17,18^. Coupled to a laptop-based bioinformatics workflow, MinION was able to detect Chikungunya virus, Ebola virus and hepatitis C virus in less than 6 hours using earlier versions of the technology^19^. A multiplex PCR method for complete on-site Zikavirus genome sequencing in samples with low viral loads has recently been developed by Quick and coworkers^20^. Partial dengue virus genomes were isothermally amplified followed by sequencing, allowing classification of strains in serotypes^21^. In veterinary virology, the use of nanopore sequencing is growing. A novel species of papillomavirus was identified in warts from giraffes, using rolling-circle amplification and nanopore sequencing^22^. The entire genome of a parapoxvirus isolated from a seal was obtained by combining data from Illumina next-generation sequencing with nanopore sequencing data^23^. One study has reported the detection of Venezuelan equine encephalitis virus from unamplified cDNA created from poly-A tailed RNA using cell culture grown viruses^24^. To the author’s knowledge, the present study is the first using MinION as an aid in porcine health management. This study was aimed to explore the possibilities of MinION as a rapid and easy-to-use diagnostic tool in pig health management for diagnosis of viral enteric disease complexes. The ability to detect high loads of cell culture-grown rotavirus and coronavirus, mimicking shedding quantities observed in diarrheic piglets, was evaluated. In a second case, the ability to detect (novel) viruses in diarrheic feces of a one-week-old piglet with diarrhea was investigated. No gene-specific or random pre-amplification of viral nucleic acids was conducted to challenge the MinION’s sensitivity. A porcine kobuvirus was discovered in the latter case and a longitudinal field study was conducted hereafter to elucidate the shedding patterns of this virus. Moreover, archival (2014) fecal samples from diarrheic suckling piglets less than two weeks old were investigated for the presence of kobuviruses, to study their epidemiology in Belgium.

## Results

### Rapid detection and characterization of cell culture-grown viruses

**Figure 1:**
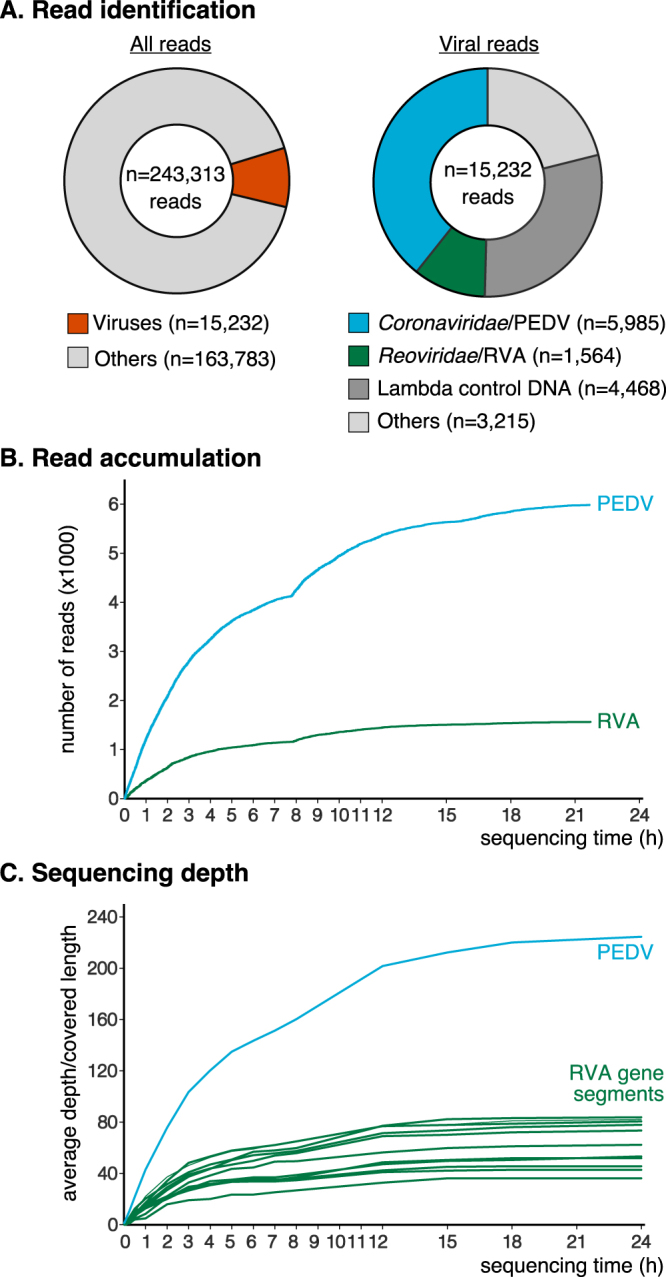
Sequencing of cell culture grown enteric viruses with MinION. (**A**) Taxonomical identification of reads. (**B**) Accumulation of porcine epidemic diarrhea virus (PEDV) and rotavirus A (RVA) reads over time. (**C**) Average sequencing depth per nucleotide position for PEDV and different RVA gene segments. Blue = PEDV, green = RVA.

Successful basecalling could be performed for 243,313 reads with a mean length of 740 nucleotides. Reads with a q-score lower than 7 were filtered out, resulting in 179,015 remaining sequences (mean length 816 nt) for use in downstream analyses. Results of the sequencing run, including taxonomical classification and mapping of reads against PEDV and rotavirus A (RVA) reference genomes are shown in Fig. 1A. After 24 hours of sequencing, a total of 15,232 reads were classified as viral by sensitive tBLASTx comparison against a complete viral database. Of these, 39.3% (n = 5,985) and 10.3% (n = 1,564) were assigned to viral families comprising *Porcine epidemic diarrhea virus* (family *Coronaviridae*) and *Rotavirus* (family *Reoviridae*, subfamily *Sedoreovirinae*), respectively. A fraction of the reads (29.3%, n = 4,468) were assigned to order *Caudovirales*. These reads originated from the lambda phage DNA used in a previous control run on the same flowcell. At 7.5 and 24.2 seconds after the start of sequencing, respectively, the first reads matching PEDV and RVA were translocated through a nanopore. Most reads were generated in the first twelve hours of sequencing and read accumulation was most exponential in the first three hours of sequencing (Fig. 1B). PEDV and RVA sequences were extracted from the dataset and mapped against viral reference genes to calculate sequencing depths over time (Fig. 1C). After one hour, sequencing depths were higher for PEDV (43.0X) than for RVA (4.9 to 22.1X). High sequencing depths were acquired after three hours of sequencing for PEDV (103.5X) and for most RVA gene segments (19.2 to 48.2X).

*De novo* assembly was executed on the quality-filtered reads prior to identification (tBLASTx) to recover viral genomes. This resulted in the recovery of the almost complete PEDV genome and RVA gene segments with identities varying between 95 and 99% compared to the reference genes (Table 1). Higher assembly accuracies (97 to 99%) were obtained when only the reads matching against rotavirus and PEDV were included for *de novo* assembly (Table 1). However, execution of *de novo* assembly prior to taxonomical classification (tBLASTx) reduced the time to identify entire viral genomes in the dataset.

**Table 1:** Comparison of different viral genomes/gene segments assembly methods.

Gene of interest	A. Canu followed by tBLASTx			B. tBLASTx followed by Canu			Size genome/segment**
	Contig Length	Reads	Identity to ref.*	Contig Length	Reads	Identity to ref.*	
PEDV	27459	3046	98%	27385	5056	98%	28033
RVA - VP1	3409	45	97%	3675	158	98%	3302
RVA - VP2	2620	109	98%	2636	186	98%	2717
RVA - VP3	2345	46	96%	2675	159	98%	2591
RVA - VP4	2318	120	99%	2328	183	99%	2359
RVA - VP6	1301	69	99%	1371	123	98%	1356
RVA - VP7	1185	45	98%	1448	70	98%	1062
RVA - NSP1	1717	42	98%	1699	112	98%	1567
RVA - NSP2	1275	32	98%	1273	66	98%	1059
RVA - NSP3	1379	31	98%	1600	67	97%	1076
RVA - NSP4	860	20	95%	877	37	98%	750
RVA - NSP5	896	22	99%	865	32	98%	664

*Identity compared to reference genome CV777 (PEDV) and 12R046 (porcine RVA).

**Based on reference strains CV777 (PEDV) and Gottfried (porcine RVA).

### Virome composition of a young diarrheic piglet using nanopore sequencing

**Figure 2:**
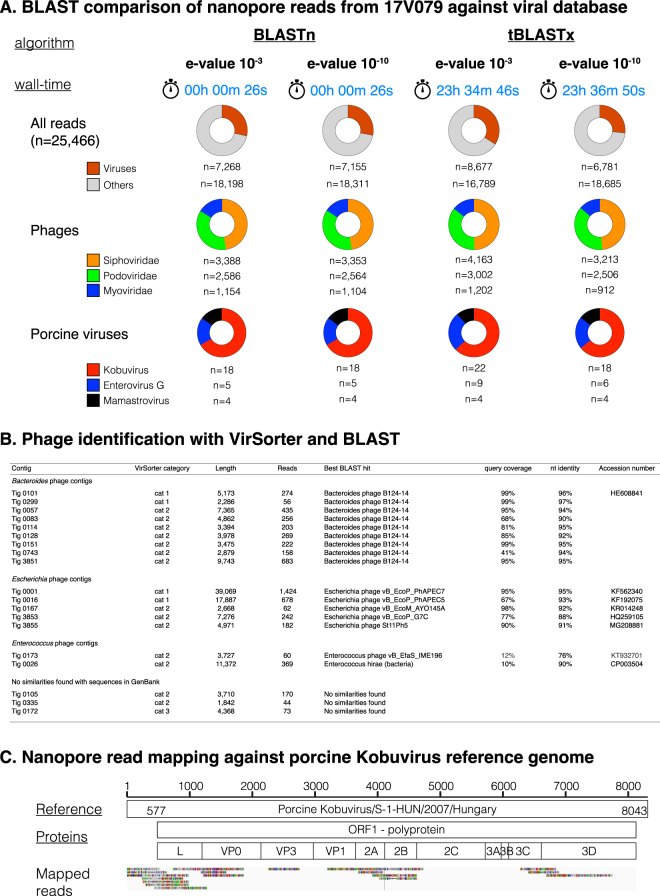
Sequencing of sample 17V079 with MinION. (**A**) Taxonomical identification of reads using BLASTn and tBLASTx. (**B**) Analysis of contigs with VirSorter and BLAST identifies several phages in the dataset. (**C**) Mapping of nanopore reads against a kobuvirus reference genome (S-1-HUN/2007; GenBank Accession Number EU787450). The clock image was licensed under Creative Commons Attribution 3.0 Unported and is free to share and remix. Attribution goes to CFCF and no changes were made.

A total of 30,088 reads were generated by sequencing the diarrheic fecal sample for three hours. Of these, 25,466 reads (q-score >7, mean read length 653 nt) were used for further analyses. Different methods were used to compare the reads against a viral database using the HPC cluster of Ghent University and results are shown in Fig. 2. Comparison against a complete viral database resulted in the detection of 6,781 to 8,677 potential viral reads, depending on the BLAST settings. BLASTn resulted in rapid taxonomical identification of reads at almost similar sensitivity compared to tBLASTx. However, there was a very high difference between wall times on the HPC cluster, with only 26 seconds of analysis time for BLASTn, versus almost 24 hours for tBLASTx. The majority of sequences were assigned to bacteriophages within the order *Caudovirales* and families *Siphoviridae* (n = 3,213 to 4,163 reads), *Podoviridae* (n = 2,506 to 3,002 reads) and *Myoviridae* (n = 912 to 1,202 reads). A *de novo* assembly was executed on the basecalled, quality filtered reads and the resulting contigs were used as input material for VirSorter analysis. Nineteen contigs were classified as sure (n = 4; category 1), somewhat sure (=14; category 2) and not so sure (=1; category 3) to be phage-like contigs (Fig. 2B). Comparison of these contigs against the GenBank database using BLAST allowed classification into four different groups. Ten contigs showed moderate to high nucleotide similarities to the *Bacteroides* phage B124-14, suggesting that they all belonged to one phage genome. This was also supported by the fact that all these contigs mapped nicely distributed across the reference genome of *Bacteroides* phage B124-14 (data not shown). The longest contig with a size of 39,069 nucleotides, together with four other contigs showed similarities (95% nt identity) to different *Escherichia* phages. As they also mapped nicely distributed across the reference genome of *Escherichia* phage vB_EcoP_PhAPEC7, it seems that they must also belong to one phage genome (data not shown). Two contigs showed poor similarity to both the *Enterococcus* phage vB_EfaS_IME_196, isolated from hospital sewage in China from an *Enterococcus faecalis* strain, and the *Enterococcus hirae* bacterial genome. The latter might be a prophage inserted in the bacterial genome. Interestingly, three contigs were identified for which no similarities were found with existing viruses in GenBank, but contig 0105 mapped to the reference genome of the *Enterococcus* phage vB_EfaS_IME196 (data not shown). These might be novel phages or divergent variants from existing phages present in GenBank.

Three eukaryotic porcine viruses, porcine kobuvirus (n = 18 to 22 reads), enterovirus G (n = 5 to 9 reads) and astrovirus (n = 4 reads) were found at much lower abundancies. The genera *Kobuvirus* and *Enterovirus* belong to the family *Picornaviridae*, whereas the genus *Mamastrovirus* belongs to the family *Astroviridae*. Kobuvirus reads were mapped against a European reference strain S-1/HUN/2007/Hungary, as shown in Fig. 2C. However, full-genome coverage at high sequencing depth was not obtained.

### Shedding of porcine kobuvirus and rotaviruses in suckling piglets

**Figure 3:**
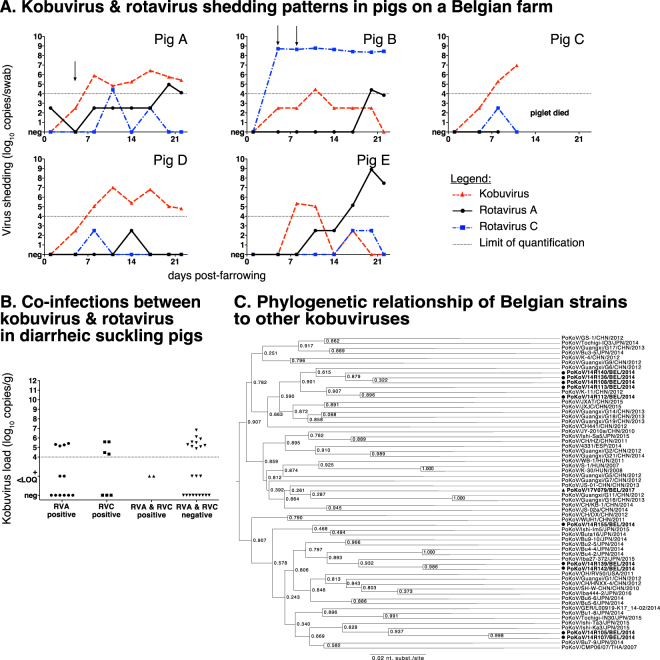
Kobuvirus epidemiology on a Belgian pig farm, in diarrheic Belgian suckling piglets and their genetic relationship to other isolates. (**A**) Shedding of rotaviruses and kobuvirus in individual suckling piglets. Episodes of diarrhea are shown with a black arrow. (**B**) Kobuvirus shedding quantities in Belgian diarrheic suckling piglets less than two weeks old in relation to the rotavirus infection status. The dotted line represents the limit of quantification. RVA = rotavirus A, RVC = rotavirus C. (**C**) Maximum-likelihood phylogenetic tree based on the 3D gene polymerase encoding gene of porcine kobuvirus with bootstrap analysis set at 500 replicates. Bootstrap values are shown in the branches of the tree. The Belgian strain 17V079, detected with MinION, is indicated with a triangle. Belgian isolates from 2014 found in diarrheic feces of suckling piglets are indicated with circles.

The shedding of porcine kobuvirus, RVA and rotavirus C (RVC) was quantitatively investigated in 5 suckling pigs of the same farm from which the diarrheic feces originated. The fecal shedding patterns of the different viruses and presence of diarrheic signs are shown in Fig. 3A. All piglets started shedding porcine kobuvirus at the end of the first week after parturition. In two piglets (A and D) the shedding was sustained and lasted for at least 2 weeks (above the limit of quantification). Peak shedding titers of the porcine kobuvirus varied between 6.42 and 7.01 log_10_ copies/swab, which is generally lower than peak shedding observed for typical enteric viruses such as rotavirus and PEDV. Moreover, the peak of shedding was not related to diarrheic episodes, questioning the role of this virus in the pathogenesis of diarrhea on the farm. Diarrheic signs were only noticed in two piglets (A and B). In piglet B, an association between high RVC shedding and diarrheic episodes was observed. In contrast, there was no direct association between peak shedding of kobuvirus and diarrheic episodes. Interestingly, a peak in kobuvirus shedding was observed in piglet C at day 11 post-farrowing. This animal died shortly hereafter, but it was unclear if this can be attributable to the kobuvirus infection. Acute RVA shedding was observed at the end of the suckling period in three of five piglets, even though all sows were vaccinated before farrowing using a bovine inactivated rotavirus vaccine.

### Retrospective analysis of porcine kobuviruses shedding in Belgian diarrheic suckling pigs and phylogenetic analysis

A total of 44 diarrheic fecal samples collected in 2014 were screened for the presence of kobuvirus using the new RT-qPCR. Of these, 25 samples (56.8%) tested positive and 18 samples showed quantifiable viral loads (4.31 to 6.83 log_10_ copies/swab). Seven samples were positive, but viral loads were too low to allow accurate quantification. The presence of RVA and RVC had been quantitatively assessed in these samples in a previous study and the occurrence of co-infections between rotaviruses and kobuvirus is shown in Fig. 3B^25^. Kobuvirus was found in equal ratios in rotavirus-negative and -positive samples. Twelve samples contained a single rotavirus infection with a high RVA load and in four of these, a high kobuvirus load (5.16 to 5.42 log_10_ copies/g) was observed. A single RVC infection was found in seven samples and in four of these tested positive for kobuvirus at high loads (4.31 to 5.59 log_10_ copies/g). A dual RVA/RVC infection was seen in two samples, but neither contained quantifiable kobuvirus loads. Many (n = 10) of the rotavirus-negative samples contained high kobuvirus loads.

Strain 17V079 showed high similarity to other Belgian porcine kobuvirus isolates from 2014 (92.1 to 94.0% nucleotide sequence identity) and the Hungarian reference strain S-1/Hun/2017 (93.4%). Furthermore, there was a high level of genetic variability between the 2014 Belgian porcine kobuvirus isolates, with nucleotide sequence identities ranging between 90.1 and 97.2%. A phylogenetic analysis, using the 3D gene of 17V079 and twelve Belgian isolates from 2014 (Fig. 3C), shows the Belgian strains clustering between strains from different geographical locations.

## Discussion

Prevention and treatment of enteric disease problems in young piglets is frequently hampered by a lack of diagnostic tools. Veterinarians are restricted to a short list of known viruses, bacteria, parasites and management factors to define a differential diagnosis. Only the most likely cause(s) of the disease will be diagnostically investigated, often leading to negative, inconclusive or incomplete results. However, metagenomics studies have indicated the existence of viral enteric disease complexes, potentially involving multiple known and novel viruses^3,4,6,7,9,10^. Detection of nucleic acids from pathogens using NGS-based metagenomics approaches is a partial solution to diagnostic testing problems and can provide a complete readout of viruses and other pathogens present in a sample. However, most NGS platforms require large investments and processing of the reads can only start at the end of the sequencing run. Viral metagenomics also requires extensive laboratory preparations, including centrifugation, filtration and nuclease treatment to discard bacterial and host nucleic acids that make up to the bulk of all nucleic acids present^13^. Furthermore, the amount of viral nucleic acids in a sample is very low, requiring targeted or random amplification of these genomes before NGS analysis. Amplification may induce bias and hampers the development of a fast diagnostic pipelines due to considerable time loss. All these factors lead to a long turnover time between sample collection and diagnostic reporting. The third-generation sequencing device MinION (ONT), holds promise as a diagnostic platform, as it allows real-time sequencing and analyses of all DNA/RNA in a sample, theoretically without needing pre-amplification of viral nucleic acids. It was the aim of the present study to evaluate this technology for use as a rapid tool for porcine viral enteric disease complex identification, without the conduction of viral nucleic acid amplification.

In a first experiment, cell culture-grown PEDV and RVA, known to induce diarrhea in young pigs, were pooled at high loads mimicking shedding quantities in diarrheic piglets. Sequencing of this pooled sample with the MinION resulted in rapid identification of both viruses. Real-time analysis of the sequencing reads was not conducted, but is achievable as previously demonstrated by Greninger and colleagues using the SURPI analysis pipeline for rapid identification of human viruses from different clinical matrices^19^. Interestingly, the first reads matching PEDV and RVA were generated respectively after 7 and 24 seconds of sequencing. High sequencing depths (43.0X) were acquired within one hour of sequencing for PEDV and within three hours for most of the eleven RVA gene segments (19.2–48.2X). Overall, higher sequencing depths were generated for PEDV that could indicate that sequencing of longer viral genomes is favored over smaller gene segments, as PEDV has a genome size of approximately 28 kb, and RVA gene segments are shorter (0.6 to 3.3 kb). This bias might have been introduced during the ligation of the sequencing adapters to the viral nucleic acids. It can be hypothesized that adapters are more easily attached to longer DNA fragments, and bias should be avoided by standardization of viral nucleic acid input length. Rapid read generation allows flexible use of the sequencing platform and sequences can be read until enough genome information of the viruses of interest is available. While the technology can be useful for giving fast readouts of viruses (<3 hours) present in a sample, thorough validation, using well-defined virus stocks, spiking experiments in matrices (e.g. feces) and real clinical samples is necessary to make sure that all members of the porcine viral enteric disease complex are accurately being diagnosed. Furthermore, the accuracy of the technology needs further improvement, as error rates of contigs from *de novo* assemblies still ranged between 1 and 5%, hindering the precise analysis of subtle but important mutations in the viral genome.

After the successful identification of the cell culture-grown viruses, the performance of the MinION was further explored by analyzing a diarrheic fecal sample of a one-week-old suckling piglet. Real-time PCR analyses were conducted for RVA, RVC, PEDV and TGEV. *Enterococcus hirae* was isolated at a private diagnostic laboratory, but this bacterial species is not considered a typical cause of diarrheic disease in pigs^26^. Viral metagenomics was conducted on this sample using the MinION and two different BLAST search algorithms were used to taxonomically identify the reads by comparing them against a complete viral database. Overall, tBLASTx with an e-value of 10^−3^ was able to identify the most viral reads compared to other search options conducted. However, BLASTn search options also reached high sensitivity, but at much lower time cost: 26 seconds instead of almost 24 hours. For rapid read analysis and searching for closely related non-divergent viral sequences, BLASTn or another fast methodology should thus be preferentially used. However, tBLASTx might pick up more divergent or novel viruses, improving overall sensitivity.

Three porcine viruses, including porcine kobuvirus, porcine mamastrovirus and enterovirus G, were identified in sample 17V079. Astro- and enteroviruses have been detected earlier in both diarrheic and non-diarrheic feces of Belgian pigs and in feces from pigs around the globe^9,10,27^. In a recent study from Thailand, the difference in prevalence of astrovirus in diarrheic (8.4%) versus non-diarrheic (4.6%) piglets less than 4-weeks-old was not statistically significant. Also other studies have shown that the role of porcine astrovirus in the pathogenesis of pig diarrhea is not completely clear^28^. In contrast, associations between diarrhea and human astrovirus infections have been made^29^. A recent study in 5 European countries (Hungary, Spain, Germany, Austria and Sweden) have indicated the widespread of porcine astroviruses in the swine population. A one hundred procent prevalence of astrovirus was found in diarrheic and non-diarrheic pigs from Austria and Spain. Porcine astroviruses have recently also been linked to outbreaks of neurological disorders in weaned piglets from Hungary, and in 5-week-old pigs and sows in the United States^30,31^. The gut might be a hypothetical entry port for such neurological astrovirus infections.

Enteroviruses have been more generally linked to neurological disorders in pigs, although they are commonly found in feces as well^11,12,32–34^. In a study from Vietnam, no significant correlation was found between diarrhea status and presence of enterovirus G in feces^35^. The involvement of both astro- and enteroviruses in the pathogenesis of enteric disorders might be questioned here, but cannot be completely ruled out. Furthermore, while sensitive tBLASTx searches were used here, there is still a possibility that a completely novel virus might be present in the dark matter of the sequencing reads. However, reporting of a porcine kobuvirus in Belgian piglets with MinION is unique. In Belgium, kobuviruses had previously only been found in diarrheic samples of calves and young cattle in Belgium^36^. In the present study, a novel RT-qPCR assay, targeting the conserved 3D gene encoding the RNA-dependent-RNA-polymerase, was developed and used to assess, for the first time, longitudinal quantitative shedding kinetics of porcine kobuvirus in pigs under field conditions. Similar kinetics were also analyzed for porcine rotavirus A and C. While suckling piglets started shedding porcine kobuvirus from one week of age, an association between peak viral shedding (6.42 to 7.01 log_10_ copies/swab) and diarrheic signs was not observed. In one pig, an association was made between diarrheic episodes and the peak of rotavirus C shedding, a well-known enteric pathogen^37,38^. Very interestingly, kobuvirus fecal loads were typically lower than those reported of well-described enteric viruses of which the pathogenicity has been proven using piglet infection models, such as PEDV and rotavirus^39–41^. Similar viral loads for porcine kobuvirus were also found in case (4.60 ± 1.76 copies/qPCR reaction) and control pigs (4.79 ± 1.72 copies/qPCR reaction) during a recent Danish study to evaluate the role of viruses in the pathogenesis of the new neonatal porcine diarrhea syndrome. The study demonstrated that kobuvirus, astrovirus, rotavirus A, porcine teschovirus, porcine norovirus and porcine coronaviruses were not involved in the pathogenesis of the syndrome^42^. The finding of low kobuvirus loads in feces casts doubt over the true enteric pathogenic tropism of the virus. Hypothetically, its replication is likely not distributed across the whole villus but limited to either enterocytes at the villus’ tips or to immune cells present in the gut. The presence of kobuvirus RNA in serum has also been demonstrated in Hungarian pigs, but it was not known if the virus is also replicating in other organs^43^. Both the oro-fecal route and the feeding of milk to sucklings pigs could be involved in virus transmission. Highest rates of infection were observed in suckling piglets, compared to older pigs, in other countries^44–46^. In our study, relatively long shedding of porcine kobuvirus was observed in three out of five animals, which may indicate that this virus may induce persistent infections. A 2011 Brazilian study demonstrated the presence of kobuvirus RNA in serum from 3-day-old piglets, which had disappeared by day 21, indicating viral clearance from the blood and excluding systemic persistence^45^. A complete lack of pathogenicity cannot be excluded, as porcine kobuviruses might play a role as a subclinically important virus. Such subclinical, yet immunosuppressive, properties have been attributed to the economically important swine pathogen porcine circovirus^47^. Of interest, one of the piglets died at the peak of kobuvirus shedding, although it was not clear if there was any causality between virus replication and the piglet’s death. *In vivo* animal experiments in a model of neonatal, conventional kobuvirus-negative piglets should be conducted to elucidate the pathogenesis of porcine kobuviruses. Attempts were made to isolate the virus in different cell lines (MA104, ST and SK), and peripheral blood mononuclear cells. There was no evidence of cytopathogenic effect after several days of incubation. Antibodies to visualize antigen expression were not available and therefore the possibility of replication without evident cytopathogenic effect cannot be ruled out. Efforts will be made to isolate the virus in porcine primary enterocyte cultures, once available.

To assess more broadly the prevalence of kobuvirus in the Belgian swine industry, a retrospective analysis of diarrheic samples from suckling piglets less than two weeks old was conducted. A high proportion (40.9%) of the samples (n = 44) contained quantifiable viral loads ranging between 4.31 to 6.83 log_10_ copies/g feces. Viral loads found were thus comparable to the loads excreted by piglets in the longitudinal analysis and the above-mentioned study from Denmark, demonstrating the endemic presence of the virus in the Belgian swine population^42^.

In the present study, non-diarrheic piglets were not included and therefore no association between kobuvirus prevalence and disease can be made. However, the prevalence of kobuvirus has been widely described in pigs from several European countries (The Netherlands (16.7%), Slovakia (63.4%), Hungary (81.0%), Czech Republic (87.3%), Austria (46.2%), Italy (52.4%), Germany (54.5%) and Sweden (45.0%)), American countries (The United States (21.9%) and Brazil (53.0%)), African countries (Kenya (14.9%) and Uganda (15.5%)) and Asian countries (Thailand (99%), South Korea (52.1%) and Vietnam (29.3%))^44–46,48–53^. In a small proportion of these studies, statistically significant associations between prevalence of kobuvirus and diarrhea in pigs were demonstrated, such as in Hungary (54.5% prevalence in healthy pigs vs 92.3% prevalence in diarrheic pigs), Spain (47.5% healthy vs 74.4% diarrheic), Brazil (41% vs 78.4%), Thailand (19.3% vs 84.5%) and Vietnam (27.6% to 40.9%)^35,45,46,52^. Indeed, it is difficult to make correlations between prevalence of the virus and diarrhea, as the pathogenicity of the virus could be largely influenced by other factors such as co-infections with other enteric viruses, microbiota and management factors.

Belgian isolates showed genetic moderate to high genetic variability, with nucleotide identities between 90.1 and 97.2%. Furthermore, they clustered diffusely between strains from different countries around the world, indicating that strains are not distinguishable based on their geographical origin.

Because most (99%) of the reads generated during sequencing of the fecal sample 17V079 matched bacteriophages upon analysis with BLAST, bacteriophages may have played an important role in the pathogenesis of the diarrheic disease. *De novo* assembled contigs were analyzed using VirSorter, a software package for mining viral signals from microbial genomic data. Such tools allow maximizing the possibility of detecting dsDNA phages^54^. Several contigs showed high similarities to the *Bacteroides* phage B124-14, found in municipal wastewater and human fecal samples. It was shown to be absent in 30 samples collected from different animal species, including pigs, and is therefore considered a human-specific phage^55,56^. The finding of several contigs, genetically similar to phage B124 and likely belonging to one phage genome, indicates that this phage found in the pig fecal sample may also replicate in the microbiome of the young pig gut and not solely in humans. However, it is possible that the phage’s replication ability in the pig’s gut is age-dependent and that very young age groups were not sampled in previous studies. Interestingly, several of the contigs found also showed similarities to *Escherichia* phages. Two of the contigs were similar to *Escherichia* phages PhAPEC5 and PhAPEC7, isolated from Belgian rivers in the neighborhood of poultry houses and known to cause lytic infections in avian pathogenic *Escherichia coli*. Electron microscopic images of the phages PhAPEC5 and PhAPEC7 indicated that they belonged to the family *Podoviridae*^57^. Two other contigs were similar to two closely related *Escherichia* phages, St11Ph5 and G7C, found in sewage and horse feces, respectively^58^. Finally, one contig showed limited similarity to an *Enterococcus* phage, isolated from hospital sewage in China, while a last contig showed moderate similaraties to the bacterial *Enterococcus hirae* genome. This region may be a prophage, inserted in the bacterial genome.

The phages found in this piglet may have reshaped the gut microbiota, allowing opportunistic bacteria such as *Enterococcus hirae* to proliferate and to start secreting toxins. It is also possible that a phage infection of bacteria in the pig’s gut led to a stress status for these bacteria, prompting the secretion of toxins. The new neonatal diarrhea syndrome described above shows high similarities to the disease described in the case 17V079 and it may be that bacteriophages are involved in the pathogenesis of this syndrome. So far, the role of phages has not been considered in the pathogenesis of several enteric disorders, but given the high abundance here, it should be in future studies.

It is clear that new technologies will change the way diagnostics are be performed in the near future. Pricing might currently be an aspect hampering high-troughput analysis of samples in swine veterinary medicine, but as the technology evolves fast, this might become very soon less relevant. Complete overviews of all viruses and other pathogens in a sample will be given in a single readout instead of requiring different diagnostic assays. However, care should be given to the interpretation of such results, as they should only be analyzed by trained veterinarians.

## Methods

### Viruses

Porcine rotavirus A (RVA) strain RVA/Pig-tc/BEL/12R046/2012/G9P[23] was isolated from a diarrheic piglet and grown for three successive passages in MA104 cells to an infectious virus titer of 10^7.8^ CCID_50_/ml. The nucleotide sequences of the 11 gene segments of this strain were resolved earlier using Sanger sequencing (GenBank accession numbers: KM82070 (VP1), KM820707 (VP2), KM827014 (VP3), KM820720 (VP4), KM820728 (VP6), KM820735 (VP7), KM820742 (NSP1), KM820672 (NSP2), KM820679 (NSP3), KM820686 (NSP4) and KM820693 (NSP5))^59^. A porcine epidemic diarrhea virus strain (PEDV, CV777) isolated in Belgium in the 1970s was adapted for growth in Vero cells in the 1980s^60^. In our Laboratory, the virus was grown to an infectious virus titer of 10^6.0^ CCID_50_/ml (GenBank accession number: AF353511).

### Origin of a fecal sample from diarrheic suckling piglets

A diarrheic fecal sample was collected from a Belgian pig on a farm housing a total of 620 sows and using a 2-week batch-production system, with a weaning age of 23 days. Topigs Norsvin sows were crossed with Piétrain boars, producing 32.6 live-born piglets per sow per year. Sow vaccinations were executed against enterotoxigenic *Escherichia coli* and *Clostridium perfringens* toxins (Suiseng, Hipra). Rotavirus A vaccination was done off-label with an inactivated bovine rotavirus A vaccine (Lactovac, Zoetis). Until recently, diarrheic problems were rarely present in suckling piglets and also very low mortality percentages (6.2–7.1%) were observed. Since the spring of 2017, enteric disease started causing more severe problems accompanied with mortality on this farm, mainly in 7-days-old suckling piglets. A diarrheic fecal sample of such a piglet was investigated at a private diagnostic laboratory (Dialab, Belsele, Belgium) and labeled 17V079. No virological cause was found to explain the diarrheic problems on the farm. The only isolated bacterium was *Enterococcus hirae*. This bacterium was thereon added to the sow vaccination schedule (inactivated autovaccine). No other pathogens were found in this sample. As the clinical picture hinted at a viral cause for the disease, the sample was sent to the Laboratory of Virology at the Faculty of Veterinary Medicine (Ghent University) for further analysis. The sample tested negative for RVA, RVC, PEDV and TGEV using in-house RT-qPCR assays^25,61,62^. Therefore, it was decided to perform a metagenomics analysis with MinION described in this study.

### Purification of viral nucleic acids

First, viral enrichment was done based on the NetoVIR protocol to obtain pure viral nucleic acids for sequencing library preparation^13^. MinION analyses of cell culture grown viruses RVA and PEDV were conducted at the Laboratory of Clinical Virology (Rega Institute, KU Leuven), whereas the diarrheic fecal sample was analyzed at the Laboratory of Virology (Faculty of Veterinary Medicine, Ghent University). RVA and PEDV stocks were centrifuged at 17,000 × *g* for 3 min. The supernatant of both suspensions was diluted to 6 log_10_ CCID_50_/ml and 500 µl of each suspension was mixed to reach an equal concentration of both viruses. This mixture was filtered using a 0.8 µm polyethersulphone filter for 1 min at 17,000 × *g*, followed by a nuclease treatment for 2 hours at 37 °C to digest free nucleic acids in the suspension: 250 µl of the sample was added to 14 µl of home-made buffer (1 M Tris, 100 mM CaCl_2_ and 30 mM MgCl_2_, pH 8), 4 µl of Benzonase Nuclease (Millipore) and 2 µl Micrococcal Nuclease (NEB) as described earlier^13^. Fourteen microliters of EDTA were added to stop the reaction, followed by extraction of nucleic acids from the viral particles using the QIAamp Viral RNA Mini Kit (Qiagen). The manufacturer’s instructions were followed but no carrier RNA was added and elution was done in 30 µl of AVE to concentrate the viral nucleic acid extract.

The diarrheic fecal sample 17V079 was processed similarly as the cell culture grown viruses, with some minor modifications. A 10% w/v suspension of the diarrhea was made in Minimum Essential Medium and centrifuged. The supernatant was filtered through a 0.45 µm syringe filter (Sarstedt) and treated with Benzonase Nuclease for 1 hour to speed up the diagnostic pipeline. Viral nucleic acids were extracted using the QIAamp Cador Pathogen Mini Kit according to the manufacturer’s instructions without addition of carrier RNA. Elution was done in a volume of 50 µl.

### cDNA and second strand synthesis for nanopore sequencing

Nucleic acids were heated at 95 °C for 2 min and chilled on ice to resolve secondary RNA structures and to denature double-stranded RNA. Superscript IV Reverse Transcriptase (ThermoScientific) was used to generate cDNA. Ten microliters of template nucleic acids were mixed with 0.5 µl random hexamer primers (Random Primer 6, New England Biolabs), 1 µl dNTP mix (NEB) and 2.5 µl nuclease-free water. Primer annealing was conducted at 65 °C for 5 min, after which 4 µl Superscript IV Reaction Buffer (ThermoScientific), 1 µl dithiothreitol (ThermoScientific) and 1 µl SuperScript IV Reverse Transcriptase (ThermoScientific) were added in a total reaction volume of 20 µl. The reaction conditions were as follows: 23 °C for 10 min, 50 °C for 10 min, 80 °C for 10 min and an infinite hold step at 10 °C.

A second strand of DNA was generated from single stranded (c)DNA molecules using the NEBNext Second Strand Synthesis Kit (NEB). Twenty microliters cDNA reaction mixture were added to 10 µl NEBNext Second Strand Synthesis Reaction Buffer, 5 µl NEBNext Second Strand Synthesis Enzyme Mix and 45 µl nuclease-free water (80 µl total reaction volume). Isothermal amplification was done at 16 °C for 1 h and double-stranded nucleic acids were purified using 144 µl of magnetic AMPure XP Beads (Beckman Coulter). Two washing steps with freshly prepared 70% ethanol were conducted before eluting in 52 µl nuclease-free water.

### Nanopore sequencing library preparation

A deoxyadenosine was ligated to the 3′-end of double-stranded nucleic acids to allow binding of complimentary sequencing adapters. Fifty microliters of (un)amplified DNA were mixed with 7 µl Ultra II End-Prep Reaction Buffer (New England Biolabs) and 3 µl Ultra II End-prep enzyme mix (New England Biolabs), and incubated at 20 °C for 5 min and 65 °C for 5 min. Next, nucleic acids were purified using 60 µl AMPure XP Beads and eluted in 31 µl nuclease-free water. Sequencing adapters, provided with the Ligation Sequencing Kit 1D (R9.4) (SQK-LSK108, ONT), were ligated to the dA-tailed nucleic acids. End-prepped DNA (30 µl) was mixed with 20 µl adapter mix (AMX, ONT) and 50 µl Blunt/TA Ligation Master Mix (New England Biolabs) in a total reaction volume of 100 µl and incubated at room temperature for 10 min. The sequencing library, containing double-stranded DNA with adapters ligated to the 3′ ends, was then purified using 40 µl AMPure XP beads. Two washing steps were conducted using 140 µl Adapter Bead Binding Buffer (ABB, ONT) before eluting in 15 µl of Elution Buffer (ELB, ONT).

### Nanopore sequencing

Two MinION flowcells (MIN-106, R9.4 chemistry) were used for these experiments. Flowcell 1 was used for the analysis of the cell culture-grown viruses and run for 24 hours. The lambda control DNA, included in the sequencing kit, was analyzed for 6 hours on this same flowcell prior to washing and loading of the PEDV/RVA sample the day after. The sample of the diarrheic piglet was analyzed on another flowcell. A priming mix containing 480 µl Running Buffer and 520 µl nuclease-free water was prepared and 800 µl was loaded on the flowcell. After five minutes, another 200 µl of priming mix was added with the SpotOn sample port in the opened position, directly before loading 75 µl sequencing library through the SpotOn sample port in a droplet manner. The library consisted of 35 µl Running Buffer (ONT), 25.5 µl Library Loading Beads (EXP-LLB001, ONT), 12 µl adapted and tethered library and 12.5 µl nuclease-free water. Sequencing was done using the software programme MinKNOW software (ONT).

### Bio-informatics analyses

Raw reads were produced by MinKNOW. Live basecalling was enabled for the first experiment using MinKNOW version 1.5.5. In the second experiment, basecalling was done after the sequencing run using Albacore (version 1.2.5., ONT). Quality scores and read lengths were visualized using NanoPlot, followed by quality filtering with NanoFilt^63^. Reads with a q-score lower than 7 were omitted. Sequences were then analyzed using different BLAST methods including BLASTn and tBLASTx (BLAST version 2.6.0; e-value cut-off 1e^−3^–1e^−10^) to compare sensitivity and run-times to detect viral sequences among the reads. A complete viral database was composed of all virus sequences in GenBank (taxonomy ID 10239, containing sequences up to 17th of September 2017). The best hit (lowest e-value) was visualized using KronaTools^64^. Reads matching viruses were extracted using Seqtk (https://github.com/lh3/seqtk) and used in downstream analyses. GraphMap (version 0.5.2) and Samtools (version 1.6) were used for mapping of reads against reference sequences, while Canu 1.6 was used for de novo assembly of viral genomes^65–68^. VirSorter was run using the ‘Viromes’ database to look for phages, with the Virome Decontamination Mode on to identify phage contigs^54^. Bio-informatics analyses were executed on a local computer cluster and the high-performance computing facilities of Ghent University. The datasets generated during and/or analysed during the current study are available from the corresponding author on reasonable request.

### Sanger sequencing of porcine kobuvirus polymerase gene

A porcine kobuvirus was discovered in the sample 17V079 using the MinION. The sequence of the 3D gene of porcine kobuvirus encodes the polymerase and is considered to be most conserved among different strains. The exact nucleotide sequence of this virus was verified using reverse transcripion polymerase chain reaction followed by Sanger sequencing, as low coverage was obtained with MinION. RT-PCR was executed using the OneStep RT-PCR Kit (Qiagen) with the newly designed primers Kobu_6049Fw and Kobu_7524Rv (IDT DNA Technologies) (Table 2). The RT-PCR reaction contained 5 µl 5 × Qiagen OneStep RT-PCR Buffer, 1 µl dNTPs, 3 µl of each primer (10 µm), 7 µl nuclease-free water, 1 µl OneStep RT-PCR enzyme mix and 5 µl template RNA or water (total reaction volume of 25 µl). RT-PCR conditions were as follows: 50 °C for 30 min, 95 °C for 15 min, followed by 30 cycles of amplification (94 °C for 30 s, 50 °C for 30 s and 72° for 90 s) and a final extension step at 72 °C for 1 min. Reactions were held at 10 °C prior to loading 5 µl PCR product with 1 µl of loading dye in a 1.5% agarose gel. Electrophoresis was conducted for 30 min at 100 V and PCR product was visualized by ethidium bromide staining and UV light. The amplicon was sent to GATC (Constance, Germany) for Sanger sequencing using an ABI 3730xl DNA Analyzer system. Quality control of the raw chromatograms was done using 4Peaks (Nucleobytes BV, The Netherlands) and BLASTn (NCBI, United States).

**Table 2:** Primers designed for porcine kobuvirus sequencing and RT-qPCR.

Application	Primer name	Sequence
RT-PCR and sequencing	Kobu_6049Fw	5′-CCTGAGATCGAGCAGTTTG-3′
	Kobu_7524Rv	5′-AAGCATGAGTCTATTCTACACA-3′
RT-qPCR	Kobu_3D_qPCR_Fw	5′-TTGGYAAYGAGACGTATGA-3′
	Kobu_3D_qPCR_Rv	5′-CCATARATCACATCATCACC-3′
	Kobu_3D_qPCR + T7_Fw	5′-TAATACGACTCACTATAGGG TTGGCAACGAGACGTATGA-3′

### RT-qPCR for porcine kobuvirus quantification in feces

Specific RT-qPCR primers (Table 2) for the porcine kobuvirus polymerase-encoding gene were designed using Primerquest and Oligoanalyzer (IDT DNA Technologies) to allow exact quantification in feces of piglets. Each RT-qPCR reaction consisted of 10 µl PrecisionPlus OneStep qRT-PCR Mastermix containing SYBR Green, ROX and an inert blue pipetting dye (Primerdesign, Southampton, United Kingdom), 0.4 µl of each primer (200 nM) and 6.2 µl nuclease-free water. Three microliters of template RNA or water were added to each tube containing 17 µl mastermix. A synthetic RNA positive control (175nt) was generated by RT-PCR using the primers Kobu3D_qPCR +T7_Fw and Kobu3D_qPCR_Rv, followed by *in vitro* transcription of this PCR product using a T7 RNA polymerase. The positive control was measured using Nanodrop and used to setup a standard curve over a linear dynamic range (LDR) from six to one log_10_ copies/reaction. Reaction conditions were as follows: 55 °C for 10 min and 95 °C for 2 min, followed by 40 cycles of denaturation (95 °C for 10 s) and annealing (58 °C for 60 s). Detection of SYBR Green fluorescence was done at the end of each annealing phase. A melt curve analysis was executed to assess specificity of the amplicons generated. Each dilution point in the standard curve and each sample was tested in duplicates. Amplicons were analyzed once on an agarose gel to assess the correct length of the amplicon and Sanger sequencing was conducted to confirm the amplification of the partial porcine kobuvirus polymerase gene. Assays were valid if the efficiency over the LDR was between 90 and 110%, and R^2^ of the standard curve replicates was >0.99. Quantification of the viral loads was possible if the Cq-values of two qPCR replicates fell within the LDR of the assay. Both replicates had to be positive for a sample to be considered as positive. If the Cq-values of specific amplicons have fallen behind the lowest point of the standard curve, the sample was considered positive but not quantifiable.

### Longitudinal investigation of kobuvirus and rotavirus shedding in suckling piglets

Upon characterization of the virome with the MinION, a longitudinal follow-up study was setup between August and September 2017. To warrant the health status of the pig stock, entrance to the farm was strictly regulated. Sampling was performed by the farmer. Detailed instructions and sampling materials were provided to the farmer. Sample collection in the longitudinal field study was done in agreement with the European legislation on animal experiments. Sample collection was approved by and done in accordance to the requirements of the Local Ethical Committee of the Faculty of Veterinary Medicine and Bioscience Engineering of Ghent University.

One day after parturition of the sows, five litters were selected at random. Within each litter, one piglet was identified for longitudinal follow-up during the entire suckling period. A dry cotton rectal swab (Copan) was collected from each individual piglet at days 1, 5, 8, 11, 14, 17, 20 and 22 after birth. The swab was placed immediately in 2 ml of viral transport medium (phosphate buffered saline containing 1000 U/ml penicillin (Continental Pharma, Puurs, Belgium), 1 mg/ml streptomycin (Certa, Braine l′Alleud, Belgium), 1 mg/ml gentamicin (Life Technologies) and 0.01% v/v Fungizone (Bristol-Myers Squibb, Braine l′Alleud, Belgium)) in a sterile 15 ml falcon tube (Sarstedt) and stored at −20 °C. Every week, samples were collected from the farm and transported to the Laboratory of Virology. The farmer was asked to mark the tube of each sample for presence or absence of diarrheic signs. Upon arrival in the Laboratory of Virology, the samples were thawed and placed on a shaker for 30 min at 4 °C to release viral particles in the transport medium. Samples were extracted using the QIAamp Cador Pathogen Mini Kit according to the manufacturer’s instructions and purified nucleic acids were eluted in 100 µl of AVE and stored at −70 °C until RT-qPCR analysis. RT-qPCR analysis was conducted, as described above, to quantify porcine kobuvirus genome copies per swab. Furthermore, RVA and RVC shedding was assessed using previously described in-house RT-qPCR assays^25,61^.

### Retrospective epidemiological and phylogenetic analysis of kobuvirus circulation in diarrheic Belgian suckling pigs

Fecal samples (n = 44) of diarrheic suckling piglets less than 2 weeks old were sent to a private laboratory by veterinarians (Dialab, Belsele, Belgium) for etiological diagnosis, as described earlier. These samples were collected in 2014 and stored at −70 °C in the laboratory. They had previously been evaluated for the presence of rotaviruses using RT-qPCR^25^. RNA extraction was conducted using the QIAamp Cador Pathogen Mini Kit (Qiagen) as described above and RT-qPCR was done to quantify the load of kobuvirus RNA copies. Samples with a quantifiable viral load were subjected to RT-PCR to amplify the 3D polymerase gene, after which Sanger sequencing was performed. The sequences encoding the polymerase of 11 Belgian porcine kobuvirus isolates were deposited into GenBank with accession numbers MH184664-MH184674. The sequences were used to conduct a multiple sequence alignment together with other porcine kobuvirus strains in MEGA 7 using the ClustalW plug-in^69^. A maximum-likelihood phylogenetic tree was constructed with RAxML using a general time reversible model with gamma distribution (20 cats, alpha: 0.121, LogLK = 14938.461) and heuristic branch swapping^70^. Tree editing was done using Affinity Designer (Serif). Pairwise distances were calculated using the p-distance model in Mega with bootstrap values set at 500 replicates.
